# Combining semi-automated image analysis techniques with machine learning algorithms to accelerate large-scale genetic studies

**DOI:** 10.1093/gigascience/gix084

**Published:** 2017-08-23

**Authors:** Jonathan A. Atkinson, Guillaume Lobet, Manuel Noll, Patrick E. Meyer, Marcus Griffiths, Darren M. Wells

**Affiliations:** 1 Centre for Plant Integrative Biology, School of Biosciences, University of Nottingham, Sutton Bonington, LE12 5RD, United Kingdom; 2 Agrosphere, IBG3, Forschungszentrum Jülich, Jülich 52425, Germany; 3 Earth and Life Institute, Université Catholique de Louvain, B-1348 Louvain-la-Neuve, Belgium; 4 InBios, Université de Liège, 4000 Liège, Belgium

**Keywords:** root, plant phenotyping, machine learning, QTL analysis

## Abstract

Genetic analyses of plant root systems require large datasets of extracted architectural traits. To quantify such traits from images of root systems, researchers often have to choose between automated tools (that are prone to error and extract only a limited number of architectural traits) or semi-automated ones (that are highly time consuming). We trained a Random Forest algorithm to infer architectural traits from automatically extracted image descriptors. The training was performed on a subset of the dataset, then applied to its entirety. This strategy allowed us to (i) decrease the image analysis time by 73% and (ii) extract meaningful architectural traits based on image descriptors. We also show that these traits are sufficient to identify the quantitative trait loci that had previously been discovered using a semi-automated method. We have shown that combining semi-automated image analysis with machine learning algorithms has the power to increase the throughput of large-scale root studies. We expect that such an approach will enable the quantification of more complex root systems for genetic studies. We also believe that our approach could be extended to other areas of plant phenotyping.

## Findings

### Background

Plant root systems have many physiological roles, including the acquisition of water and nutrients, making them of critical importance for yield establishment in crops. The improvement of root architectural traits will thus be crucial in delivering the yield improvement required to ensure future global food security [1, 2]. Unfortunately, root systems are difficult to analyse and quantify: they are intrinsically complex due to their highly branched tree structure [3], and their growth in an opaque medium (soil) makes them difficult to observe.

For many years, root researchers have used specific experimental setups to observe and quantify root system architecture. Among these, the “pouch system” is widely used by the community to acquire large numbers of images of root systems [4–6]. In this approach, plants are grown on the surface of paper, allowing the root system to be imaged. The analysis of the resulting root images can be performed either using semi-automated [7, 8] or fully automated root image analysis software [9, 10]. Semi-automated tools require input and validation by an expert user to faithfully extract the geometry of the root system. However, such user interaction is time consuming, which can strongly hinder the application of these approaches to large datasets (such as those required for quantitative genetic studies). Fully automated software tools are faster, but the extracted descriptors are prone to unexpected errors and the quantified traits are usually less informative [3]. This has led to image analysis being described as a new “bottleneck” in plant phenotyping [11].

Machine learning (a multidisciplinary field of computer science, statistics, artificial intelligence, and information theory) encompasses a range of techniques for the automatic production of analytical models and has been attracting the interest of the plant science community in recent years. Machine learning is breaking new ground in plant science via the automation of procedures and experiments that previously required manual curation. These automated workflows are catalysing the development of new data-driven plant science [12], including remote sensing [13], species identification [14], and phenotyping [15–18]. Recently, a new approach utilizing machine learning algorithms has been proposed for the identification of root system architectural traits; a Random Forest model was trained on corresponding ground-truth and image descriptors. The resulting trained model was used to analyse a new set of simulated images and was shown to be much more accurate than the direct image descriptors [3].

Here, we have evaluated this technique using a similar approach with experimental images and assessed its application to a large-scale genetic study. Our rationale was 2-fold. First, we can reasonably expect a certain level of homogeneity within datasets coming from a single genetic screening, as root systems from a given species share common attributes. Second, semi-automated root image analysis tools can be used to extract the ground-truth on a subset of images. Such ground truths can be used to train a machine learning algorithm that can then be used to analyse the remaining images in the dataset.

We show that such an approach (i) can yield better results than fully automated software analysis, (ii) is time-efficient compared to performing a semi-automated analysis on the whole dataset, and (iii) is able to correctly identify previously found quantitative trait loci (QTL) for root traits.

### Overview of the analysis workflow

**Figure 1:**
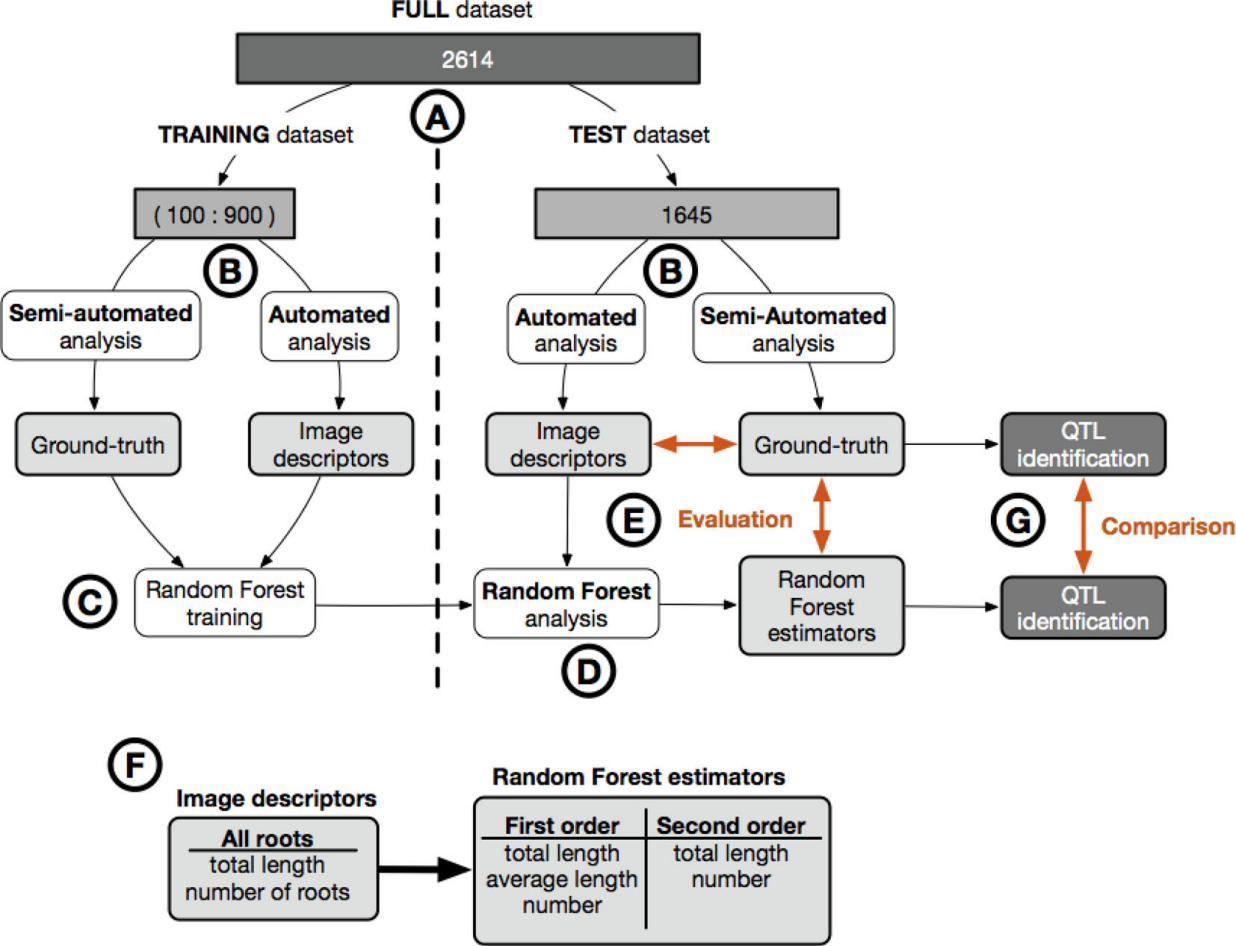
Overview of the analysis pipeline used in this study. (**A**) We divided the full dataset (2614 images) into two: a training set (100 to 900 images) and a test set (1645 images). (**B**) For each dataset, all the images were analysed using a semi-automated root image analysis tool (RootNav) to extract the ground-truth, as well as with a fully automated root image analysis tool (RIA-J) to extract image descriptors (see the text for details). (**C**) We trained a Random Forest model on the image descriptors and the ground-truth from the training dataset. (**D**) We applied the Random Forest model on the image descriptors from the test dataset. (**E**) We compared the image descriptors and the Random Forest estimators from the test dataset with their corresponding ground-truth. (**F**) Comparison of biologically relevant metrics extracted with the automated analysis and the Random Forest analysis. (**G**) QTL were identified and compared using both Random Forest estimators and the ground-truth data.

The dataset consists of 2614 RGB images of the roots of wheat seedlings obtained from growth pouch experiments as detailed in Atkinson et al. [5]. The images are of the root systems of the parental lines and 94 members of the Savannah × Rialto doubled haploid mapping population, with a minimum of 15 replicates per line. All images were processed using the semi-automatic root analysis tool RootNav (RootNav; RRID:SCR_015584) [7] to extract phenotypic root system architectural traits, representing the “ground-truth” values. A subset of these images has been previously used to identify QTL for root traits [5]. The dataset was divided in 2 (Fig. 1A): a training dataset, *Dtrain*, of variable size (between 100 and 900 images out of 969) and a test dataset, *Dtest*, of 1645 images, comprising the image set used to identify QTL in the original experimental work [5]. For all images, we first extracted the true values of the traits (ground-truth; *Ttrain, Ttest*) using RootNav [7]. We then extracted a k-dimensional feature vector (“the image descriptor”) of each image using a fully automated analysis pipeline, RIA-J [3]; the corresponding sets are *Itrain, Itest* (Fig. 1B). We used the extracted data (*Itrain, Ttrain*) to train a Random Forest model M: *I → T* to predict the different ground-truths based on the image descriptors (Fig. 1C and F) [3]. The trained Random Forest model **M** was then applied to the image descriptors *Itest* from the test dataset *Dtest* to predict the ground-truth *Ttest* (named Random Forest estimators) (Fig. 1D). The accuracy of both the image descriptors and the Random Forest estimators were then compared to the ground-truth acquired with RootNav.

One of the aims of our analysis was to assess the minimal size required for a training dataset. Therefore, we used different numbers of images for training: 100, 200, 300, 400, 500, 600, 700, 800, and 900. For each set, we randomly selected the images out of the 969 images that comprised the training dataset, then repeated the training/accuracy procedure described above. To account for the fact that the images were randomly selected, for each test size, we repeated the procedure 10 times.

For each training dataset size, we used the Random Forest estimators to detect QTL regions associated with the different traits quantified (Fig. 1G). The identified QTL regions were then compared to those previously identified using RootNav, as well as those identified using the direct image descriptors.

### Random Forest estimators have a greater accuracy and greater biological relevance than image descriptors

It has been previously shown that Random Forest estimators are better at predicting the ground-truth values of various root system metrics compared to direct image descriptors [3]. However, this evaluation used simulated images, rather than a “real” experimental dataset.

**Figure 2:**
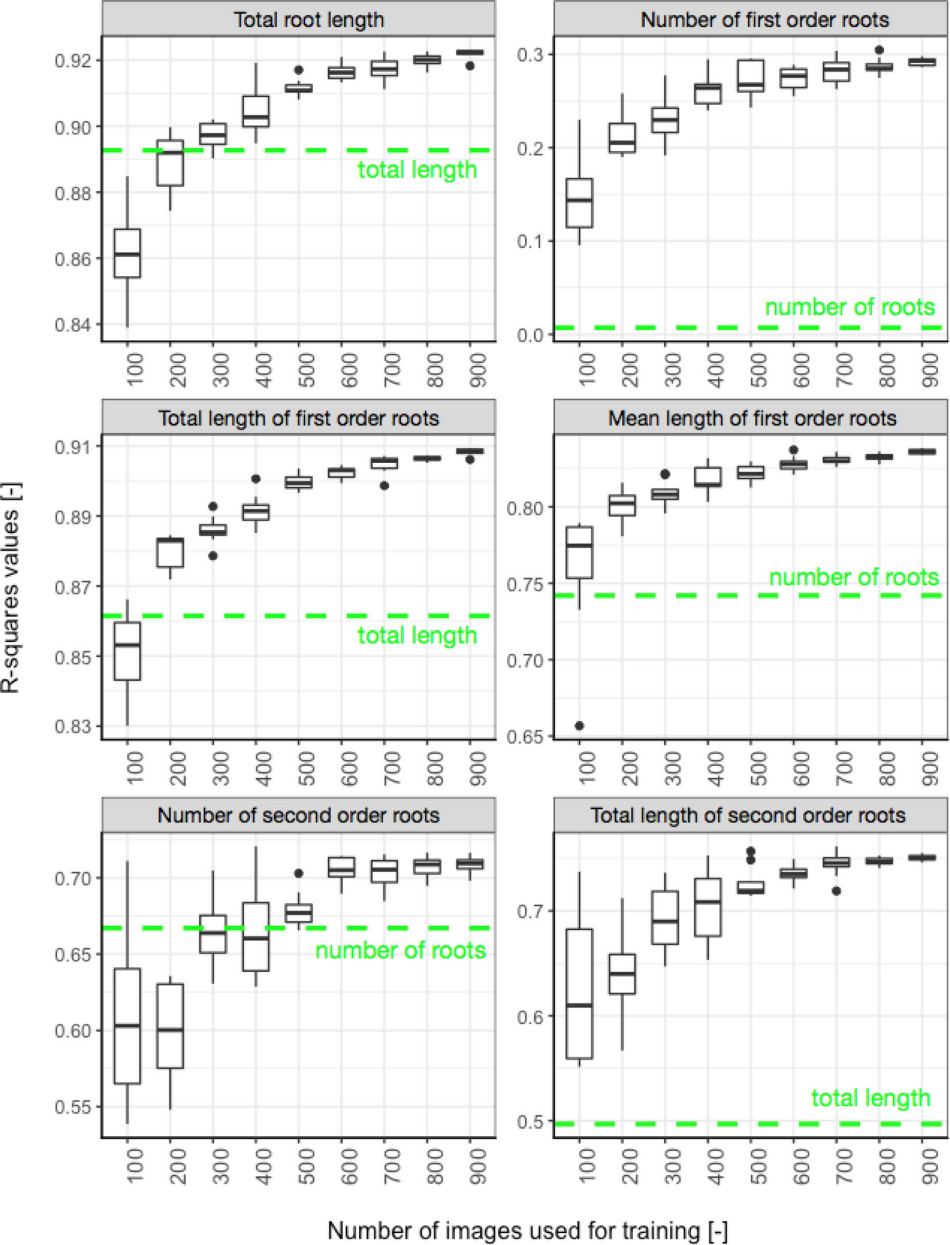
Accuracy of the Random Forest estimators. The *R*^2^ values of the linear regression between the Random Forest estimators and the ground-truths were computed for each size and repetition of training datasets. The dotted line represents the *R*^2^ value between the most closely related image descriptors and the ground-truth.

Here we show that this approach can also be used with experimental data yielding better results than the direct image descriptors (Fig. 2). We also show that, as expected, increasing the size of the training dataset increases the accuracy of the estimated metrics. For our data, we observe a strong increase in accuracy up to a dataset size of 500 training images, after which the improvement becomes marginal. Our approach also allows for the prediction of new metrics, not obtained using the direct image descriptors. For instance, the direct descriptors do not differentiate between the different root orders, whereas the Random Forest model does.

We observed a decrease in the variability of the predicted values as the number of training images increased. This may be the result of a greater accuracy of the prediction, but may also be due to the fact that the same images are randomly selected for each repetition. As the number of training images increases, we expect the number of identical images across repetitions to increase as well (the total number of images in the training set being 969).

### Random Forest estimators identify the correct QTLs

Plant phenotyping studies often use mapping populations to dissect the genetic architecture of complex traits by identifying regions of chromosomal DNA that correlate with phenotypic variation, termed quantitative trait loci. The images in our test dataset were used in such a study to identify several QTL for root traits in wheat seedlings [5]. In addition to testing the accuracy of the Random Forest approach in estimating root system parameters, we wanted to know if these parameters could be used reliably for the identification of QTL. Since QTL identification had already been performed on our test dataset, we could directly assess the performance of our new pipeline against the original approach by using the same QTL detection technique on both the direct image descriptors and the traits derived from the random forest models.

The Random Forest models, trained on different numbers of images (100:900), were used on the image descriptors from the test dataset to predict 9 estimator datasets (named EST-100 to EST-900) for use in the QTL analysis (see Table 1). This was done to assess the minimum size for the training dataset required for reliable QTL detection, which may be lower than that required to accurately predict the trait values themselves. The R package R/qtl [19] was used for QTL detection on the image descriptor dataset and the 9 Random Forest predicted datasets [5]. Identified QTL were then directly compared to those found in this paper.

**Table 1:** Results from the QTL comparison for the different estimator datasets

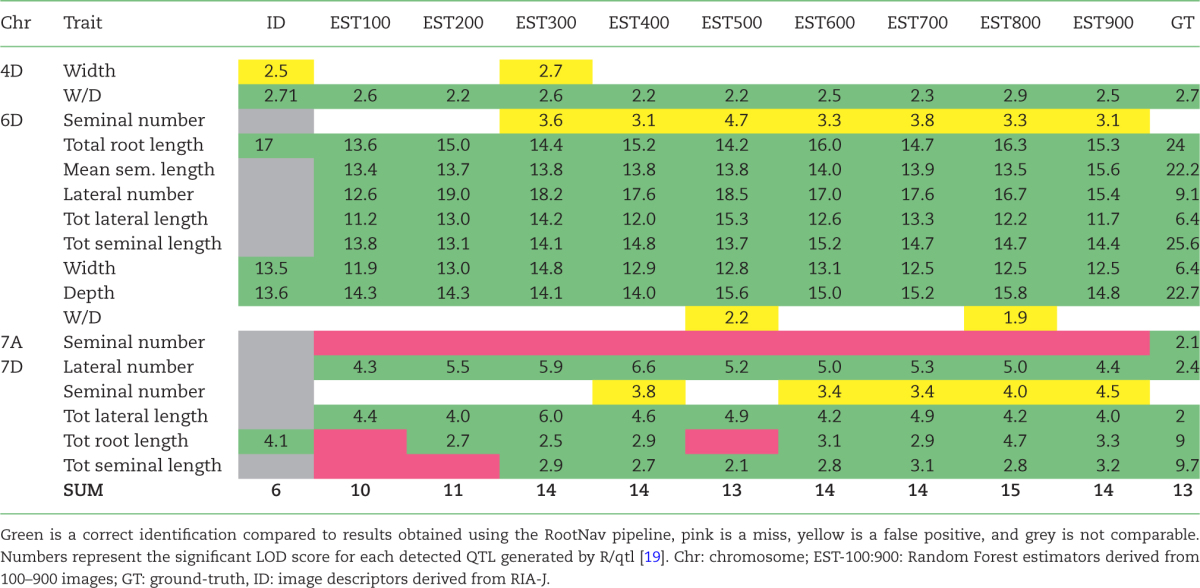											

We observed that 12 out of 13 of the expected QTL were correctly identified using the estimators from the Random Forest models trained on 600 or more images (EST-600: EST-900). We also observed that even using the smallest training set of 100 images (EST-100), most of the QTLs were identified (10/12), with 12 of 13 being identified with the estimators from the model trained with 300 images (EST-300). We did not observe an increase in the logarithm of odds (LOD) score with the increase of images (Table 1).

In addition, 4 extra QTL were identified on chromosomes 4D and 6D. Two of these were identified for width and width-depth ratio from EST-300, EST-500, and EST-800 datasets (Table 1). Although in this example these have been labelled as false positives as they were not detected in the original study, they both have related QTL co-localizing in the same positions (the 4D width QTL co-localizes with a W/D QTL, and the 6D W/D QTL co-localizes with both a width and depth QTL at the same location). Both QTL were also found using the image descriptors utilized to train the Random Forest model, possibly explaining their identification. Two additional QTL for seminal (first-order) root number were identified on chromosomes 6D and 7D from the EST-300 to EST-900 datasets. This is most likely due to the inability of the Random Forest to accurately predict the seminal number in our dataset (*R*^2^ < 0.3) (Fig. 2).

In the majority of cases, the identified QTL had the same confidence intervals and similar peak marker positions as previously reported for all Random Forest models. Interestingly, the 4D QTL had a very similar confidence interval (position 0.8–67.6 previously reported vs 0–67.6 here), but a different peak marker position (position 4.8 previously reported vs position 30–34 here). It was also noted that lateral root QTL found on 7D had a reduced confidence interval compared to those previously reported (positions 0–101.8 previously vs 0–62.4 here).

### Combining semi-automated analysis and machine learning techniques increases the throughput of our image analysis pipeline

Extracting meaningful information from images of root systems is a subjective, tedious, and often time-consuming process. As a general rule, automated techniques can only extract a limited amount of biologically relevant metrics and are often limited to young plants. Semi-automated tools are able to extract more metrics and with a greater accuracy, but at the expense of user interaction time (which makes them unsuited for large-scale genetic studies). As a result, large genetic screens targeting root system traits often focus on a set of simple traits that can be automatically extracted.

Here we have shown that machine learning techniques can be used to automatically extract a large set of root system metrics. To train the machine learning algorithm on our dataset, we estimated that 600 root images are needed. Additional images are needed to validate the accuracy of the machine learning estimators (around 100). These images have to be traced with a semi-automated tool to extract the parameters in the first place. Thus, instead of tracing all the images (in our case about 2600), only a subset (700) was needed. It was previously estimated that tracing 1 image takes, on average, 2 minutes. In our case, the whole dataset would represent a workload of 87 hours. With the combined pipeline, the workload decreased to 23 hours (27%).

**Figure 3:**
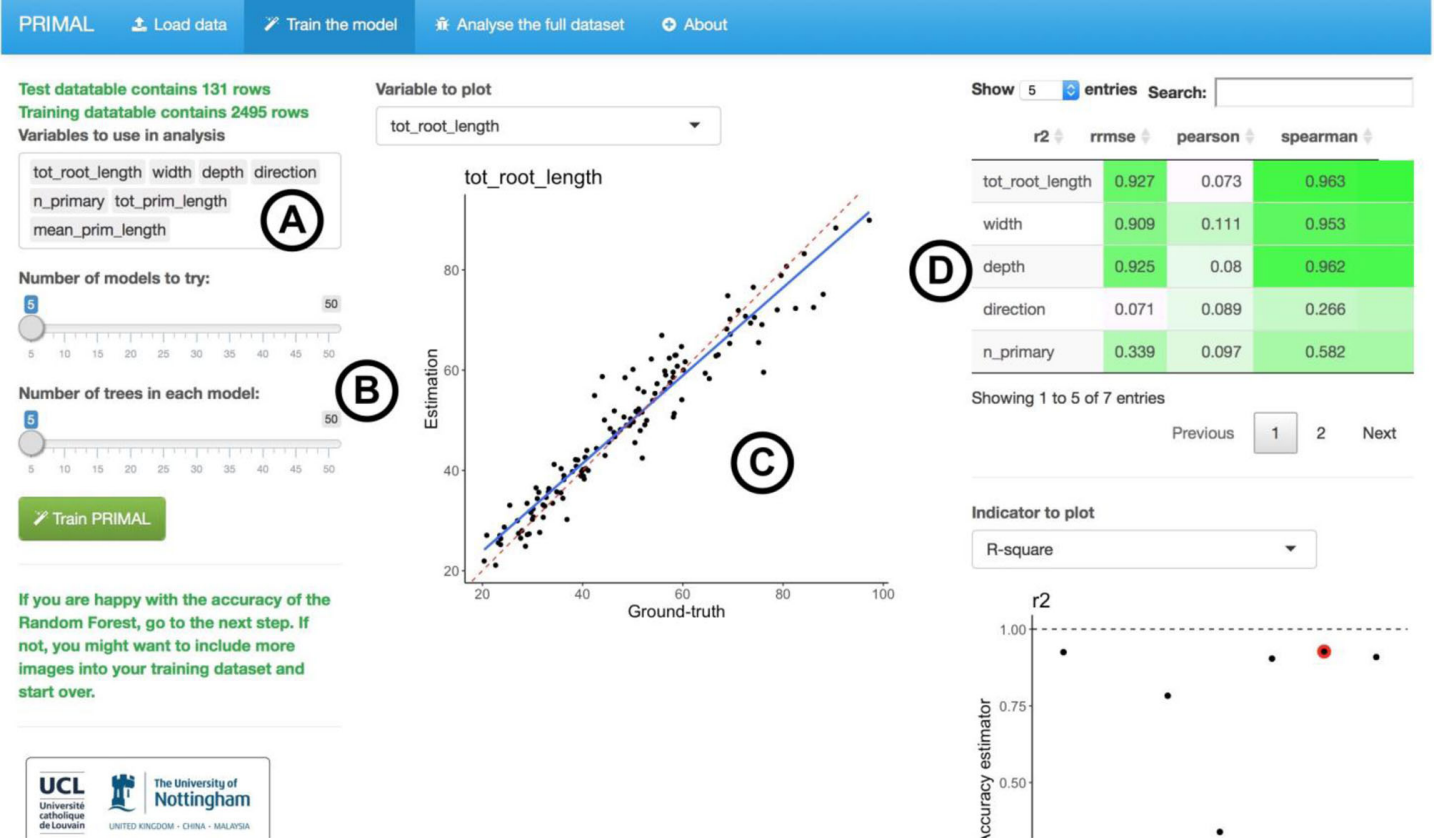
Screenshot of PRIMAL. (**A**) Variable to evaluate with the Random Forest algorithm. (**B**) Random Forest algorithm parameters. (**C**) Visualization of the accuracy of the Random Forest estimators. (**D**) Accuracy metrics for the different descriptors.

In this example, we used a published dataset, for which the ground-truth data were already available [5]. In order to easily apply this approach to future studies, we have created the R application Pipeline of Root Image analysis using MAchine Learning (PRIMAL) (Fig. 3) [20]. We recommend the following analysis strategy:

1. Use a fully automated tool to extract image descriptors for the entire dataset.
2. Use a semi-automated tool to extract the ground-truth for 200 random images (the GROUND-TRUTH DATASET). Remove these images from the global dataset.
3. Use PRIMAL to train the Random Forest model and analyse the data.
4. Check the accuracy of the prediction of the Random Forest model. If the prediction is not satisfactory, increase the number of images in the ground-truth dataset and repeat the procedure. The final number of images in the ground-truth dataset will vary between experiments, plants, conditions, etc. In our example, 500 images were needed to reach a satisfactory accuracy for the Random Forest predictions.

A detailed version of this protocol is available at protocols.io [21].

It should be noted that the prediction accuracy of the Random Forest estimation is highly dependent on the homogeneity of the data. For example, a Random Forest model trained on maize root systems will most likely fail when applied to wheat. However, for large-scale genetic studies, where only 1 species is used in the analysis, this should not be an issue. The accuracy of the Random Forest estimators is also a function of the variability of the direct descriptors in the dataset. Using a large set of descriptors that better discriminate the different images might help increase the accuracy of the Random Forest descriptors.

## Conclusions

Genetic studies on root architecture require large annotated datasets of biologically relevant traits. Automated analysis tools can be used to extract descriptors from large libraries of root images. Unfortunately, these descriptors are prone to error, and their biologically relevancy is not always clear. Alternatively, semi-automated tools enable the retrieval of more precise architectural traits, but, due to the required skilled user inputs, they are often unsuitable for large datasets.

Here, we used a Random Forest model to predict architectural traits based on automatically extracted image descriptors. The model was trained on a subset of the whole dataset that had been previously analysed using a semi-automated tool. This strategy allowed us to (i) decrease the time required for the analysis by 73% (compared to the semi-automated analysis of the whole dataset) and (ii) accurately predict meaningful architectural traits.

In order to make our pipeline available to the community, we have created an application, available at [20].

## Methods

A detailed version of the protocol described here is available at protocols.io [21].

## Availability of supporting source code and requirements

— Project name: PRIMAL, Pipeline of Root Image analysis using MAchine Learning
— Project home page: https://plantmodelling.github.io/primal/
— Operating system(s): platform independent
— Programming language: R
— Other requirements: none
— License: GPL

## Availability of supporting data

The following supporting data are open and available from the *GigaScience* repository, *Giga*DB [22]:

1. Root system image dataset #1. Images of root systems of plants tagged with genotype information; 1665 images from [5].
2. Root system image dataset #2. Training images without genotype information; 969 images.
3. Root System Markup Language files for both image datasets.
4. Full genotype mapping information for the population, from CerealsDB: http://www.cerealsdb.uk.net/cerealgenomics/CerealsDB/Excel/SavannahXRialto_map_data_May_2013.xls.

## Abbreviation

QTL: quantitative trait locus; LOD: logarithm of odds.

## Competing interests

At the time of publication, G.L. was a Guest Editor for the *GigaScience* thematic series “Plant Phenomics.”

## Funding

This work was supported by the Biotechnology and Biological Sciences Research Council [grant number: BB/D019613/1]; the European Research Council [grant number: 294729]; and the Fonds De La Recherche Scientifique [grant number: 1.A.320.16F].

## Author contributions

Conceptualization: G.L., D.M.W., J.A.A.; formal analysis: G.L., J.A.A., M.N., P.E.M.; resources: J.A.A., M.G., M.N., P.E.M.; writing—original draft: G.L., J.A.A.; writing—review and editing: G.L., J.A.A., M.G., D.M.W., M.N.; visualization: G.L., J.A.A.

## Supplementary Material

GIGA-D-17-00146_Original-Submission.pdf

GIGA-D-17-00146_Revision-1.pdf

GIGA-D-17-00146_Revision-2.pdf

Response-to-Reviewer-Comments_Original-Submission.pdf

Response-to-Reviewer-Comments_Revision-1.pdf

Reviewer-1-Report-(Original-Submission).pdf

Reviewer-2-Report-(Original-Submission).pdf

## References

[1] Herder GD, Van Isterdael G, Beeckman T The roots of a new green revolution. Trends Plant Sci 2010;15:600–7. 20851036 10.1016/j.tplants.2010.08.009

[2] Lynch JP Turner review no. 14. Roots of the second green revolution. Aust J Bot 2007;55:493–512.

[3] Lobet G, Koevoets IT, Noll M Using a structural root system model to evaluate and improve the accuracy of root image analysis pipelines. Front Plant Sci 2017; doi: 10.3389/fpls.2017.00447. 10.3389/fpls.2017.00447 PMC5376626 28421089

[4] Hund A, Trachsel S, Stamp P Growth of axile and lateral roots of maize: I. Development of a phenotying platform. Plant Soil 2009;325:335–49.

[5] Atkinson JA, Wingen LU, Griffiths M Phenotyping pipeline reveals major seedling root growth QTL in hexaploid wheat. J Exp Bot Soc Experiment Biol 2015;66:2283–92. 10.1093/jxb/erv006 PMC4407652 25740921

[6] Gioia T, Galinski A, Lenz H *GrowScreen*-*PaGe*, a non-invasive, high-throughput phenotyping system based on germination paper to quantify crop phenotypic diversity and plasticity of root traits under varying nutrient supply. Funct Plant Biol 2016;44(1):76–93. 10.1071/FP16128 32480548

[7] Pound MP, French AP, Atkinson JA RootNav: navigating images of complex root architectures. Plant Physiol 2013;162:1802–14. 23766367 10.1104/pp.113.221531 PMC3729762

[8] Lobet G, Pagès L, Draye X A novel image-analysis toolbox enabling quantitative analysis of root system architecture. Plant Physiol Am Soc Plant Biol 2011;157:29–39. 10.1104/pp.111.179895 PMC3165877 21771915

[9] Leitner D, Felderer B, Vontobel P Recovering root system traits using image analysis exemplified by two-dimensional neutron radiography images of lupine. Plant Physiol 2014;164:24–35. 24218493 10.1104/pp.113.227892 PMC3875805

[10] Cai J, Zeng Z, Connor JN RootGraph: a graphic optimization tool for automated image analysis of plant roots. EXBOTJ 2015;66:6551–62. 10.1093/jxb/erv359 PMC4623675 26224880

[11] Minervini M, Scharr H, Tsaftaris SA Image analysis: the new bottleneck in plant phenotyping [applications corner]. IEEE Signal Process Mag 2015;32:126–31.

[12] Ma C, Zhang HH, Wang X Machine learning for big data analytics in plants. Trends Plant Sci 2014;19:798–808. 25223304 10.1016/j.tplants.2014.08.004

[13] Ali I, Greifeneder F, Stamenkovic J Review of machine learning approaches for biomass and soil moisture retrievals from remote sensing data. Remote Sensing 2015;7:16398–421.

[14] Babatunde OH, Armstrong L, Leng J A computer-based vision systems for automatic identification of plant species using kNN and genetic PCA. J Ag Inform 2015; doi:10.17700/jai.2015.6.1.152.

[15] Minervini M, Abdelsamea MM, Tsaftaris SA Image-based plant phenotyping with incremental learning and active contours. Ecol Inform 2014;23:35–48.

[16] Singh A, Ganapathysubramanian B, Singh AK Machine learning for high-throughput stress phenotyping in plants. Trends Plant Sci 2015;21:1–15. 10.1016/j.tplants.2015.10.015 26651918

[17] Weiss U, Biber P, Laible S Plant species classification using a 3D LIDAR sensor and machine learning. In: Ninth International Conference on Machine Learning and Applications, Washington, DC, 2010, pp. 339–45. New York, NY: IEEE, 2011.

[18] Pound MP, Atkinson JA, Burgess AJ Deep Machine Learning provides state-of-the-art performance in image-based plant phenotyping. Gigascience 2017:6(9):1–7. doi: 10.1093/gigascience/gix083. 10.1093/gigascience/gix083 PMC5632296 29020747

[19] Broman KW, Wu H, Sen S R/qtl: QTL mapping in experimental crosses. Bioinformatics 2003;19:889–90. 12724300 10.1093/bioinformatics/btg112

[20] PRIMAL Pipeline of root image analysis using MAchine learning. https://plantmodelling.github.io/primal/. Accessed 10 August 2017.

[21] Guillaume Lobet PRIMAL: Pipeline of root image analysis using machine learning. protocols.io 2017 http://dx.doi.org/10.17504/protocols.io.h7bb9in.

[22] Atkinson JA, Lobet G, Noll M Supporting data for “Combining semi-automated image analysis techniques with machine learning algorithms to accelerate large-scale genetic studies.” GigaScience Database 2017 http://dx.doi.org/10.5524/100346. 10.1093/gigascience/gix084 PMC5632292 29020748

