# Erratum to: Combining semi-automated image analysis techniques with machine learning algorithms to accelerate large-scale genetic studies

**DOI:** 10.1093/gigascience/giy043

**Published:** 2018-07-20

**Authors:** Jonathan A Atkinson, Guillaume Lobet, Manuel Noll, Patrick E Meyer, Marcus Griffiths, Darren M Wells

In the final publication of “Combining semi-automated image analysis techniques with machine learning algorithms to accelerate large-scale genetic studies,” by Jonathan Atkinson A. et al., the first column heading for Table 1 was incorrect.

Furthermore, the publication month for reference 18 was incorrect. The heading and reference 18 have been corrected and the corrected manuscript is published in *GigaScience*, Volume 6, Issue 10: https://doi.org/10.1093/gigascience/gix084.
