# Supplementary material for: Combining semi-automated image analysis techniques with machine learning algorithms to accelerate large-scale genetic studies
Source: Gigascience. 2017 Aug 23;6(10):1–7. doi: 10.1093/gigascience/gix084 (PMC5632292; doi:10.1093/gigascience/gix084)
Supplement: Response-to-Reviewer-Comments_Original-Submission.pdf [file gix084_Response-to-Reviewer-Comments_Original-Submission.pdf]

Author's Response To Reviewer Comments

Please see attached "Response to Reviews".
