# Supplementary material for: Combining semi-automated image analysis techniques with machine learning algorithms to accelerate large-scale genetic studies
Source: Gigascience. 2017 Aug 23;6(10):1–7. doi: 10.1093/gigascience/gix084 (PMC5632292; doi:10.1093/gigascience/gix084)
Supplement: Response-to-Reviewer-Comments_Revision-1.pdf [file gix084_Response-to-Reviewer-Comments_Revision-1.pdf]

Author's Response To Reviewer Comments

All editor revisions have been followed in the revised version of the manuscript (attached). We have also added the spreadsheet of mapping data to P11L24 as an item available from GigaDB.
