# Supplementary material for: Combining semi-automated image analysis techniques with machine learning algorithms to accelerate large-scale genetic studies
Source: Gigascience. 2017 Aug 23;6(10):1–7. doi: 10.1093/gigascience/gix084 (PMC5632292; doi:10.1093/gigascience/gix084)
Supplement: Reviewer-1-Report-(Original-Submission).pdf [file gix084_Reviewer-1-Report-(Original-Submission).pdf]

Reviewer Report

Title: Combining semi-automated image analysis techniques with machine learning algorithms to accelerate large scale genetic studies

Version: Original Submission **Date:** 7/12/2017

Reviewer name: Noah Fahlgren

Reviewer Comments to Author:

The manuscript "Combining semi-automated image analysis techniques with machine learning algorithms to accelerate large scale genetic studies" by Atkinson and colleagues presents an extension of a machine learning method they originally developed using a synthetic root image dataset. In this study, the Random Forest approach was used on an experimental dataset of wheat root images. The overall approach is to build a trainable model to estimate biologically-relevant root system characteristics using automatically extracted features (descriptors). In this way, root system features that are high-quality, but time consuming to collect by hand, can be estimated using features that can be extracted relatively easily using automated computational approaches. The authors explore several interesting questions for using a machine learning approach: 1) how much training data is needed?; 2) how accurate are results derived from the trained model versus those derived from automatically extracted metrics?; 3) how do QTL for traits identified by the gold-standard method (semi-automated analysis) compare to those identified by the Random Forest approach? The manuscript demonstrates that a relatively small amount of training data (700 images out of a dataset of 2600) can be used to automate the remaining full dataset analysis with similar accuracy to slow, semi-automated approaches, and better accuracy than automated approaches. In addition to these results, the authors provide a number of tools to enable similar analysis by other groups, including an R package for implementing the approach (PRIMAL) and a protocol for collecting the necessary training data at protocols.io.Minor comments:1. I found some of the terminology used confusing at first and suggest that these be described (maybe with examples) more in the introduction. In particular, I did not immediately recognize the difference between the types of outputs from semi-automated versus fully-automated root image analysis approaches. These end up being referred to as "ground-truth" and "descriptors," respectively. When I looked at the sample datasets in the PRIMAL GitHub repository I think I understand what these refer to now. The "ground-truth" measurements are biological root system measurements like total root length, number of primary roots, number of secondary roots, etc. Versus "descriptors" refer to indirect measurements from images such as total area, convex hull area, etc.2. The paragraph on page 5, lines 27-31 is a little unclear to me. Does this mean that the variability in measured values between different repetitions decreased as the training dataset got larger (for the posited reasons)?3. For the recommended strategy to analyze a new dataset (page 9, line 28) is the suggestion to use 100 random images for semi-automated analysis (ground-truth) just a recommended starting point? Just trying to reconcile this number with the analysis of training set size presented in the manuscript.

Level of Interest

Please indicate how interesting you found the manuscript: An article of importance in its field

Quality of Written English

Please indicate the quality of language in the manuscript: Acceptable

Declaration of Competing Interests

Please complete a declaration of competing interests, considering the following questions:

- Have you in the past five years received reimbursements, fees, funding, or salary from an organisation that may in any way gain or lose financially from the publication of this manuscript, either now or in the future?
- Do you hold any stocks or shares in an organisation that may in any way gain or lose financially from the publication of this manuscript, either now or in the future?
- Do you hold or are you currently applying for any patents relating to the content of the manuscript?
- Have you received reimbursements, fees, funding, or salary from an organization that holds or has applied for patents relating to the content of the manuscript?
- Do you have any other financial competing interests?
- Do you have any non-financial competing interests in relation to this paper?

If you can answer no to all of the above, write 'I declare that I have no competing interests' below. If your reply is yes to any, please give details below.

I declare that I have no competing interests

I agree to the open peer review policy of the journal. I understand that my name will be included on my report to the authors and, if the manuscript is accepted for publication, my named report including any attachments I upload will be posted on the website along with the authors' responses. I agree for my report to be made available under an Open Access Creative Commons CC-BY license (http://creativecommons.org/licenses/by/4.0/). I understand that any comments which I do not wish to be included in my named report can be included as confidential comments to the editors, which will not be published.

I agree to the open peer review policy of the journal
