# Supplementary material for: Combining semi-automated image analysis techniques with machine learning algorithms to accelerate large-scale genetic studies
Source: Gigascience. 2017 Aug 23;6(10):1–7. doi: 10.1093/gigascience/gix084 (PMC5632292; doi:10.1093/gigascience/gix084)
Supplement: Reviewer-2-Report-(Original-Submission).pdf [file gix084_Reviewer-2-Report-(Original-Submission).pdf]

Reviewer Report

Title: Combining semi-automated image analysis techniques with machine learning algorithms to accelerate large scale genetic studies

Version: Original Submission **Date:** 7/20/2017

Reviewer name: Roman Garnett

Reviewer Comments to Author:

This manuscript considers the problem of so-called "semi-automated image analysis" for studying plant root systems in the presence of large amounts of data. The idea is to gather ground-truth data on a small subset of the available data (at high cost), then use these data to train a machine-learning model (here, random forests), which is used to predict the phenotypes of interest on the rest of the data. The authors perform a small study where they show that this protocol can achieve good performance at relatively low cost compared to exhaustive analysis, and that the predictions made by the machine learning model are high-fidelity, both in terms of predictive power and also in terms of their ability to identify QTLs. The authors provide open-source software implementing their pipeline on GitHub.I am a strong believer in machine learning (it is my main research focus), and I agree entirely with the argument presented by the authors. Machine learning can help manage large-scale datasets (becoming increasingly more common) while avoiding unreasonable (often simply unavailable or impossible to attain) human effort for obtaining ground truth.Unfortunately, I believe several important details are missing from the manuscript as it stands; the story is not entirely complete for the reader (at least this reader). The "Overview of the analysis workflow" section begins describing "the dataset," but at this point in the paper, I do not yet know what the dataset is. After a couple lines I can surmise the dataset comprises 2614 images, split into 969 training images and 1645 test images. We are told that the test images are winter wheat images (page 4 line 21, p4l21). Are the training images from the same dataset, or are they from a different source? In p4l22, we understand that "ground truth" (what?) is extracted from these images using software, but beyond piecing together that the ground truth is presumably root phenotypes, I don't know what is meant by this statement. Only from examining Figures 1 and 2 can I find (at least a subset?) of the phenotypes of interest. Similarly, on p4l23, "image descriptors" are extracted using off-the-shelf software. What is the nature of these descriptors? What is the dimensionality? Are there parameters for the RIA-J algorithm(s) that are important? It would be very helpful to plainly clarify details such as these.By the bottom of page 4, I have pieced together most of a typical machine-learning pipeline: we extract features for all the data, train a model (random forest) given the features and ground-truth labels for a training subset (969 images of something), then predict these values on the test set (1645 images of winter wheat). However, at the top of page 5, and throughout the rest of the paper, it seems like the test set disappears entirely. Can the authors please explain why we suddenly choose a new test set by sampling from the training set? And the phrase "959 images that comprised the test dataset" on p5l3? I had a very difficult time understanding this setup and why it would be adopted. It is certainly not standard practice in machine learning, and it raises the question of where the 1645 test images went and why they were discussed at all. A held-out test

dataset would avoid the issues discussed at the bottom of page 5, for example.What is the meaning of "...both the direct image descriptors and the traits derived from the random forest models" in p6l10? Previously "image descriptors" meant features extracted from images from RIA-J, but now I can only assume the authors mean the ground-truth extracted using RootNav.Minor notes:- There is an easy fix for the issue described in p7l2: take the median prediction of the random forest members rather than the mean.- The authors seem to be performing a linear regression based on the random-forest predictions. This is a bit odd and not standard practice when using random forest models. Can you motivate/defend this choice?In addition to missing some key details, the paper could use some minor editing. I list a few (nonexhaustive) corrections below.Overall, this seems like a nice contribution that would be of interest to GigaScience readers. I would like to see some additional details and clarification of some key points before publication.Minor corrections:- author list: footnotes are properly typeset after punctuation (in this case, commas)- p2l3: This would be easier to parse if root system were hyphenated (root-system)- p2l8: automatically-extracted -> automatically extracted- p2l12 and elsewhere: no need to capitalize quantitative trait loci- p2l16: in large scale -> of large-scale- p2l18: area -> areas- p3l18: such approaches -> these approaches (to avoid three instances of "such")- p3l24: I'm not sure machine learning is an "emerging" field; it started in the 1950s.- p3l29: data driven -> data-driven- p4l11: ground-truths -> ground truth- p9l15: Machine Learning -> machine learning- p9l27-30: spacing problems at beginning of items- p9l27: extract image descriptors global dataset -> extract image descriptors for the entire dataset(?)

Level of Interest

Please indicate how interesting you found the manuscript: An article whose findings are important to those with closely related research interests

Quality of Written English

Please indicate the quality of language in the manuscript: Needs some language corrections before being published

Declaration of Competing Interests

Please complete a declaration of competing interests, considering the following questions:

- Have you in the past five years received reimbursements, fees, funding, or salary from an organisation that may in any way gain or lose financially from the publication of this manuscript, either now or in the future?
- Do you hold any stocks or shares in an organisation that may in any way gain or lose financially from the publication of this manuscript, either now or in the future?
- Do you hold or are you currently applying for any patents relating to the content of the manuscript?

- Have you received reimbursements, fees, funding, or salary from an organization that holds or has applied for patents relating to the content of the manuscript?
- Do you have any other financial competing interests?
- Do you have any non-financial competing interests in relation to this paper?

If you can answer no to all of the above, write 'I declare that I have no competing interests' below. If your reply is yes to any, please give details below.

I declare I have no competing interests

I agree to the open peer review policy of the journal. I understand that my name will be included on my report to the authors and, if the manuscript is accepted for publication, my named report including any attachments I upload will be posted on the website along with the authors' responses. I agree for my report to be made available under an Open Access Creative Commons CC-BY license (http://creativecommons.org/licenses/by/4.0/). I understand that any comments which I do not wish to be included in my named report can be included as confidential comments to the editors, which will not be published.

I agree to the open peer review policy of the journal
